# The *in silico* identification and characterization of a bread wheat/*Triticum militinae* introgression line

**DOI:** 10.1111/pbi.12610

**Published:** 2016-09-16

**Authors:** Michael Abrouk, Barbora Balcárková, Hana Šimková, Eva Komínkova, Mihaela M. Martis, Irena Jakobson, Ljudmilla Timofejeva, Elodie Rey, Jan Vrána, Andrzej Kilian, Kadri Järve, Jaroslav Doležel, Miroslav Valárik

**Affiliations:** ^1^ Institute of Experimental BotanyCentre of the Region Haná for Biotechnological and Agricultural ResearchOlomoucCzech Republic; ^2^ Munich Information Center for Protein Sequences/Institute of Bioinformatics and Systems BiologyInstitute for Bioinformatics and Systems BiologyHelmholtz Center MunichNeuherbergGermany; ^3^ Division of Cell BiologyDepartment of Clinical and Experimental Medicine, Bioinformatics Infrastructure for Life SciencesLinköping UniversityLinköpingSweden; ^4^ Department of Gene TechnologyTallinn University of TechnologyTallinnEstonia; ^5^ Diversity Arrays Technology Pty LtdCanberraACTAustralia

**Keywords:** GenomeZipper, alien introgression, comparative analysis, chromosome rearrangement, chromosome translocation, linkage drag

## Abstract

The capacity of the bread wheat (*Triticum aestivum*) genome to tolerate introgression from related genomes can be exploited for wheat improvement. A resistance to powdery mildew expressed by a derivative of the cross‐bread wheat cv. Tähti × *T. militinae* (*Tm*) is known to be due to the incorporation of a *Tm* segment into the long arm of chromosome 4A. Here, a newly developed *in silico* method termed rearrangement identification and characterization (RICh) has been applied to characterize the introgression. A virtual gene order, assembled using the GenomeZipper approach, was obtained for the native copy of chromosome 4A; it incorporated 570 4A DArTseq markers to produce a zipper comprising 2132 loci. A comparison between the native and introgressed forms of the 4AL chromosome arm showed that the introgressed region is located at the distal part of the arm. The *Tm* segment, derived from chromosome 7G, harbours 131 homoeologs of the 357 genes present on the corresponding region of Chinese Spring 4AL. The estimated number of *Tm* genes transferred along with the disease resistance gene was 169. Characterizing the introgression's position, gene content and internal gene order should not only facilitate gene isolation, but may also be informative with respect to chromatin structure and behaviour studies.

## Introduction

Using interspecific hybridization to widen a crop's gene pool is an attractive strategy for reversing the genetic bottleneck imposed by domestication and for compensating the genetic erosion, which has resulted from intensive selection (Feuillet *et al*., 2008). Much of the pioneering research in this area has focused on bread wheat (*Triticum aestivum*), in which over 50 related species have been exploited as donors thanks to the plasticity of the recipient's genome (Jiang *et al*., 1993; Wulff and Moscou, 2014). Typically, introgression events have involved the transfer of a substantially sized donor chromosome segment, which, along with the target, probably bears gene(s), which impact negatively on the host's fitness (a phenomenon also called ‘linkage drag’) (Gill *et al*., 2011; Qi *et al*., 2007; Zamir, 2001). For this reason, very few introgression lines are represented in commercial cultivars (Rey *et al*., 2015). The prime means of reducing the length of an introgressed segment is to induce recombination with its homoeologous region (Niu *et al*., 2011). The success of this strategy is highly dependent on the conservation of gene content and order between the donor segment and its wheat equivalent.

The level of resolution with which introgression segments can be characterized has developed over the years along with advances in DNA technology. Large numbers of genetic markers have been identified in many crop species, including wheat (Bellucci *et al*., 2015; Chapman *et al*., 2015; Sorrells *et al*., 2011; Wang *et al*., 2014). In a recent example, a wheat mapping population has been genotyped with respect to >100 000 markers, but the mapping resolution achieved has only enabled the definition of around 90 mapping bins per chromosome (Chapman *et al*., 2015). Given that the genomes of most donor species are poorly characterized, marker data at best allow only the position of an introgressed segment to be defined on the basis of the loss of wheat markers; they cannot determine either the size of the introduced segment or analyse its genetic content. The recently developed ‘Introgression Browser’ (Aflitos *et al*., 2015) combines genotypic data with phylogenetic inferences to identify the origin of an introgressed segment, but to do so, a high‐quality reference sequence of the host genome is needed, along with a large set of donor sequence data. The first of these requirements is being addressed by a concerted effort to acquire a reference sequence for bread wheat (www.wheatgenome.org). So far, only chromosome (3B) has been fully sequenced, and the gene content of each wheat chromosome has been obtained (Choulet *et al*., 2014; IWGSC, 2014). The so‐called GenomeZipper method (Mayer *et al*., 2011), based on a variety of resources, has been used to predict gene order along each of the 21 bread wheat chromosomes (IWGSC, 2014).

The improved resistance to powdery mildew of an introgressive line 8.1 derived from the cross of bread wheat cv. Tähti (genome formula ABD) and tetraploid *T. militinae* (*Tm*; genome formula A^t^G) is known to be mainly due to the incorporation of a segment of *Tm* chromatin containing the resistance gene *QPm‐tut‐4A* into the long arm of chromosome 4A (Jakobson *et al*., 2006, 2012). Here, a novel *in silico‐*based method, termed rearrangement identification and characterization (RICh), has been developed to identify the sequences suitable for generating markers targeting an introgression segment such as the one from *Tm*. The method integrates the GenomeZipper approach with shotgun sequences of chromosome with the introgression. The RICh method was also effective in confirming the identity of the chromosomal rearrangements, which occurred during the evolution of modern wheat.

## Results

### Chromosome sorting, sequencing and assembly

The flow karyotype derived from the DAPI‐stained chromosomes of the DT4AL‐TM line included a distinct peak (Figure S1) corresponding to the 4AL telosome (4AL‐TM), which enabled it to be sorted to an average purity of 86.2%. The contaminants in the sorted peak comprised a mixture of fragments of various chromosomes and chromatids. DNA of all 45 000 sorted 4AL‐TM telosomes was amplified by DNA multiple displacement amplification (MDA). To minimize the risk of representation bias, the products from three independent amplification reactions were pooled. From the resulting 4.5 μg DNA, a total of ~6.2 Gb of sequence was obtained, which was subsequently assembled into 279 077 contigs of individual length >200 bp, with an N50 of 2068 bp (Table 1). When the assembly was aligned with the reference genome sequences of *Brachypodium distachyon* (Vogel *et al*., 2010), rice (IRGSP, 2005) and sorghum (Paterson *et al*., 2009), it was apparent that the 4AL‐TM telosome shares synteny with segments of *B. distachyon* chromosomes Bd1 and Bd4, rice chromosomes Os3, Os6 and Os11 and sorghum chromosomes Sb1, Sb5 and Sb10 (Figure S2).

**Table 1:** Assembly statistics of chromosome arms 4AL‐TM, 4AS‐CS and 4AL‐CS

	4AS‐CS	4AL‐CS	4AL‐TM
Sequencing read depth	241x	116x	23x
Total contigs	301 954	362 851	279 077
Total bases (bp)	282 335 959	361 971 522	266 737 930
Assembly coverage^a^	0.89x	0.67x	0.49x
Min contig length (bp)	200	200	200
Max contig length (bp)	70 057	129 043	28 604
Average contig length (bp)	935	998	956
N50 length (bp)	2782	3053	2068

The data for 4AS‐CS and 4AL‐CS arms are taken from IWGSC (2014) and data for 4AL‐TM were acquired in this study.

a The size of chromosome arms 4AS‐CS (318 Mbp) and 4AL‐CS (540 Mbp) were taken from Šafář *et al*. (2010). To estimate the assembly coverage of the 4AL‐TM arm, the 4AL‐CS size was used.

### Origin of the introgression segment

**Figure 1:**
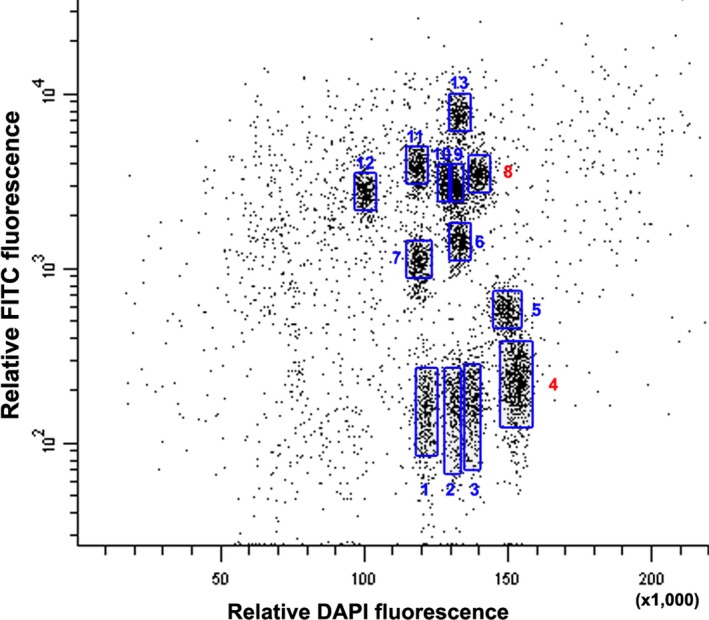
The bivariate flow karyotype of *T. militinae*. Mitotic chromosomes at metaphase were stained with DAPI and GAA microsatellites were labelled with FITC. A set of 13 distinct clusters were obtained (shown boxed). Cluster #8 harbours the *Tm* chromosome (7G) which was the origin of the introgression segment present in line 8.1. Cluster #4 harbours a putative homoeolog of 7G and based on its width and shape most likely comprises a mixture of two distinct chromosomes.

The chromosomal origin of the *Tm* introgression segment was established by initially flow sorting the *Tm* chromosome complement. This was achieved by pretreating the chromosomes with fluorescence *in situ* hybridization in suspension (FISHIS) (Giorgi *et al*., 2013) in which GAA microsatellites were fluorescently labelled by FITC. The resulting DAPI *vs* GAA bivariate flow karyotype succeeded in defining 13 distinct clusters (Figure 1). As the haploid chromosome number of *Tm* is 14, one of the clusters was therefore deemed likely to harbour a mixture of two distinct chromosomes. Two of the clusters (#4 and #8) contained sequences that were amplified by the *Xgwm160* (Roder *et al*., 1998) and *owm82* primers (these two markers are linked to the *QPm‐tut‐4A* gene from *Tm* introgression). The dispersed profile of cluster #4 (Figure 1) suggested that it was composed of two different A^t^ genome chromosomes, because all G chromosomes were identified due to a higher GAA content (Badaeva *et al*., 2010). The *owm72* marker, also linked to the *QPm‐tut‐4A* gene, amplified two fragments in *Tm*, one of size 205 bp and the other of size 250 bp; only the former was amplified from 4AL‐TM telosome or of cluster #8. The fluorescence *in situ* hybridization (FISH) profile of the chromosomes present in cluster #8 unambiguously identified the introgressed segment as deriving from chromosome 7G.

### GenomeZipper improvement

A chromosome 4A zipper was constructed based on Chinese Spring (CS) chromosome specific survey sequences (CSSs) using 1780 specific DArTseq markers ordered in consensus genetic map (Table S1). As DArTseq marker sequences are short (69 nt) and generally nongenic, they were initially anchored to the CSS assembly; this step reduced the number of useful markers to 632 (CSS‐DArTseq markers), of which 102 mapped to the short arm and 530 to the long arm. The first version of the zipper comprised a total of 2398 loci. The resulting model for 4AS was collinear with Bd1, Os3 and Sb01, as reported previously (Hernandez *et al*., 2012). However, the one for 4AL was a mosaic of 15 orthologous blocks (based on the rice genome as the reference), derived from Os11/Bd4/Sb5, Os3/Bd1/Sb1 and Os6/Bd1/Sb10 (Figure S3a). Validation for this complex structure was sought from analysis of the subset of 2638 SNP loci (Wang *et al*., 2014), which had been assigned a bin locations based on an analysis of a panel of established 4A deletion lines (Endo and Gill, 1996): of these, 750 mapped to five deletion bins on 4AS and 1888 to 13 deletion bins on 4AL (Figure S3, Balcárková *et al*., unpublished). The analysis allowed 329 SNP loci (113 on 4AS, 216 on 4AL) to be integrated into the new 4A zipper. Of the 113 4AS SNP loci, just four mapped to an inconsistent locations, demonstrating the model's accuracy; however, six (#3, #6, #8, #12, #14 and #16) of the 15 4AL blocks were inconsistent with respect to the multiple SNP loci allocations. For example, block #12—positioned in the subtelomeric region according to the zipper—included 18 SNP loci assigned to the pericentromeric region. The GenomeZipper was therefore rerun after first removing the 62 CSS‐DArTseq markers associated with the misassignment of the blocks (Table S2); of the 570 CSS‐DArTseq markers retained (Table S3), 79 were anchored to at least one of the *B. distachyon*, rice or sorghum scaffolds. The set of 2132 loci (745 on 4AS and 1387 on 4AL) revealed just six (rather than 16) blocks (Figure S3b, Table S2). The final structure resembles that described by Hernandez *et al*. (2012). When the model was retested with SNP markers, no further discrepancies were flagged along distal part of chromosome arm 4AL (Figure S3b).

### The *in silico* characterization of the evolutionary chromosome rearrangements on 4AL

**Figure 2:**
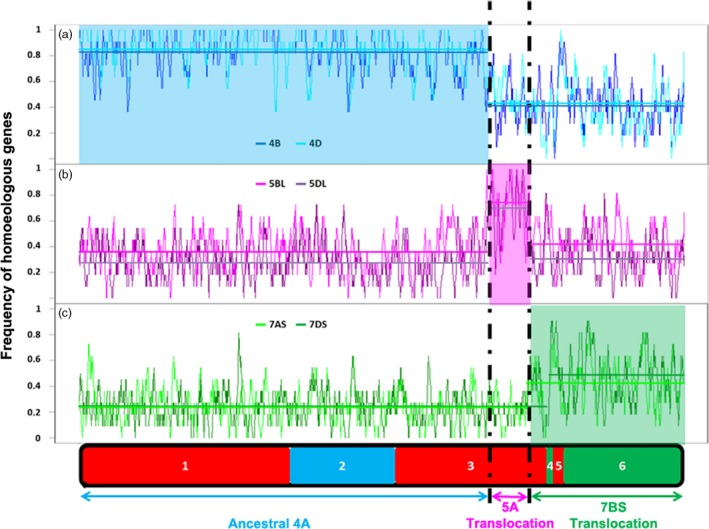
Variation in homoeologous gene density along the various 4A‐CS chromosome segments compared to their homoeologous chromosomes. The structure of native 4A‐CS chromosome is represented at the bottom with syntenic blocks with rice genome shown in different colours (red = Os3; blue = OS11; green = Os6). (a) The 4A homoeologous gene density compared to 4B and 4D chromosomes, (b) comparison with the 5BL and 5DL chromosome arms and (c) comparison with chromosome arms 7AS and 7DS is shown as homoeologous genes frequency histogram. Homoeologous regions are characterized by a high average frequency (denoted by the horizontal lines). The lower average frequency shown by the group 7 chromosomes reflects a significantly lower sequencing coverage.

The RICh method is based on a stringent identification and density estimation of homoeologs and is validated using a segmentation analysis. To test the approach, the CSS‐based scaffolds of chromosome arms 4BS, 4BL, 4DS, 4DL, 5BL, 5DL, 7AS and 7DS (IWGSC, 2014) were compared with that of chromosome 4A, applying as the criteria a 90% level of identity and a minimum alignment length of 100 bp. The numbers of homoeologous loci obtained were, respectively, 719, 762, 636, 877, 850, 673, 602 and 627 (mean 718), but no common distinct blocks allowing for the definition of evolutionary translocations could be identified. A window size of eleven genes was then selected from the 4A zipper for the subsequent segmentation analysis. The ancestral 4AS and 4AL arms had an average density of 0.83, while the remainder of 4AL had a density of only 0.41 (Figure 2a). 4BL and 4DL sequences were homologous to 4AS, and 4BS and 4DS ones to 4AL, confirming the pericentromeric inversion event uncovered before (Devos *et al*., 1995; Hernandez *et al*., 2012; Ma *et al*., 2013; Miftahudin *et al*., 2004). Immediately following the ancestral 4AL region, the density of homoeologs associated with chromosome group 5 increased (5BL and 5DL: 147 genes, density 0.73), identifying the presence of ancestral 5AL chromatin on this arm (Figure 1b). Finally, the most distal segment of 4AL was associated with an increased density of chromosome group 7 (7AS and 7DS: 557 genes, density 0.45), confirming the ancestral translocation event involving 7BS (Figure 1C).

### Characterization of the *Tm* introgression segment

**Figure 3:**
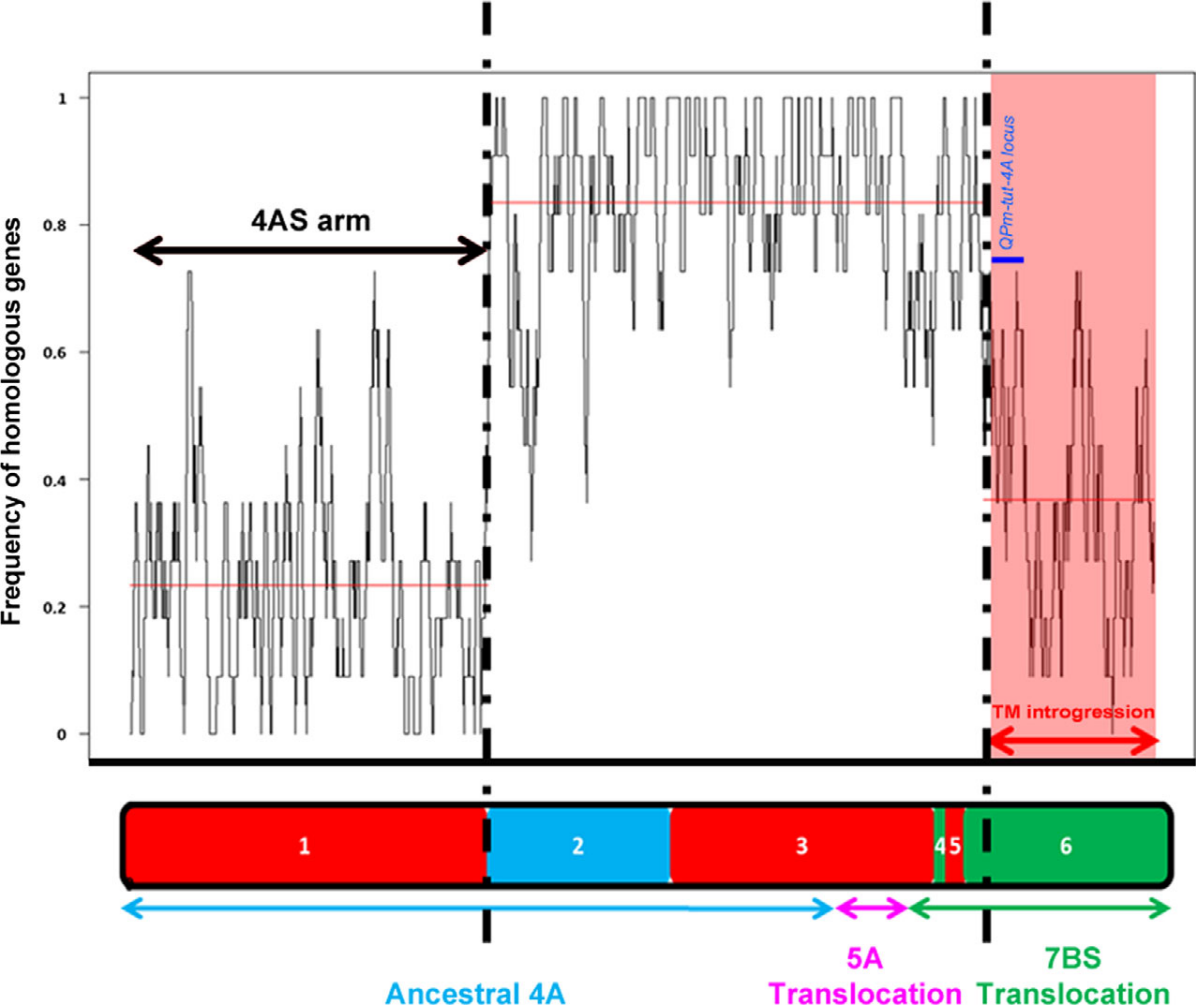
Variation in homologous gene density between 4A‐CS chromosome and 4AL‐TM telosome. The structure of native 4A‐CS chromosome is represented at the bottom with syntenic blocks with rice genome shown in different colours (red = Os3; blue = OS11; green = Os6). The homologous gene density along the 4A‐CS zipper compare to 4AL‐TM assembly is shown with the black line. The segment of the *Tm* introgression overlaps the 7BS translocation in 4AL (red highlight). The equivalent region on the 4AL‐CS telosome harbours 357 genes, only 131 have homologous genes on the *Tm* segment. The dark blue bar represents approximate localization of the *QPm‐tut‐4A* locus.

The RICh approach was then used to characterize the 4AL introgressed *Tm* segment. A direct comparison between the 4AL‐TM sequence assembly and the 4A‐CS zipper (95% identity, 100 bp minimum alignment length) was then made. For the long arm, the segmentation analysis revealed two distinct regions (Figure 3): the more proximal one had a high density of homologous genes (~0.84, 863 loci), so likely corresponds to a region of the 4AL telosome inherited from bread wheat (Figure 3). However, in the distal part of the arm, the homologous gene density fell to ~0.37, suggesting this as the site of the translocation event (Figure 3). Considering the same number of genes in the homologous regions of CS DT4AL chromosome arm (4AL‐CS) and 4AL‐TM, the comparison between these proximal segments revealed that 16% of homologous genes (167 of 1030) in the 4AL‐TM assembly were not identified and may be accounted to the sequencing and assembly imperfection. If this rate of imperfection is applied to the regions including the introgressed segment (357 CS genes *vs* 131 *Tm* homologous genes), the presence of 169 CS nonhomologous genes in the introgression segment could be estimated. The number of such genes represents the size of linkage drag (neglecting allelic variation of the homologous genes).

## Discussion

Introgression from related species provides many opportunities to broaden the genetic base of wheat, but its impact on wheat improvement has been limited by a combination of imperfect homology between donor and recipient chromatin, the loss of key recipient genes, the suppression of recombination and linkage drag effect. Thus, obtaining an accurate understanding of the size, homology, orientation and position of an introgressed segment could help to determine which introgression events are more likely to avoid incurring a performance penalty. Such knowledge would also be informative in the context of isolating a valuable gene introduced via an introgression event. Gaining this information requires saturating the target region with molecular markers. In an effort to clone of *QPm‐tut‐4A* gene introgressed to the wheat 4A chromosome from *T. militinae,* we developed new method for chromosome rearrangements and introgressions identification and characterization.

The presence of ancient intra‐ and interchromosomal rearrangements is a known complicating issue in the polyploid wheat genome, and the 4AL chromosome arm, which is one of the site of the introgression event selected in line 8.1, has a particularly complex structure. The composition of the proximal segment of the 4AL telosomes carried by DT4AL‐TM and the standard CS DT4AL stock was largely identical, as expected. However, distal part of the telosomes differs in presence of *Tm* introgressive segment (Jakobson *et al*., 2012), but no difference by synteny blocks could be detected. In hybrids between the tetraploid forms *T. turgidum* and *T. timopheevi*, Gill and Chen (1987) noted that while the latter's G genome chromosomes paired most frequently with those from the B genome, chromosome 4A was occasionally involved in pairing with chromosome 7G, presumably as a result of the presence of the 7BS segment on the *T. turgidum* 4AL arm. The likelihood is therefore that the *Tm* chromosome 7G segment, which has contributed the 4A‐based powdery mildew resistance of line 8.1, was introduced via homologous recombination with the segment of 4AL carrying 7BS chromatin.

To increase resolution of the analysis, the GenomeZipper method (Mayer *et al*., 2011), combining genetic maps, data from chromosome shotgun sequencing, and synteny information with sequenced model genomes has been adopted. The method has been useful for developing virtual gene orders in both wheat and barley chromosomes (IWGSC, 2014; Mayer *et al*., 2011). The most crucial data set is a reliable genetic map, which serves as backbone to integrate and orient the identified syntenic blocks. Two zippers for chromosome 4A have been published to date. The first was based on relatively low coverage sequencing of the chromosome, employing as its backbone a barley linkage map formed from expressed sequence tags distributed over the chromosome arms 4HS (117 loci), 4HL (16 loci), 5HL (36 loci) and 7HS (36 loci) (Hernandez *et al*., 2012). The second was based on the 4A CSS and wheat SNP map and consisted of 167 markers on 4AS ordered into 56 mapping bins and 200 (92 mapping bins) markers on 4AL; these were combined with a linkage map developed from a mapping population bred from a cross between bread wheat cv. Opata and a synthetic wheat (Sorrells *et al*., 2011). Neither of these two zippers was able to provide a sufficient level of resolution to identify the *Tm* introgression into 4AL chromosome arm. The present new zipper was based on consensus DArT map derived from crosses with CS and comprised 55% more markers and 25% more mapping bins than the latter one, which approximately doubled the number of ordered genes/loci (2132 *vs* 1004), and was informative with respect to the *Tm* introgression. When this improved zipper was used in conjunction with the RICh method, it was also possible to recognize the three evolutionary rearrangements, which have long been known to have generated the structure of the modern chromosome arm 4AL (Figure 2) (Devos *et al*., 1995; Hernandez *et al*., 2012; Ma *et al*., 2013; Miftahudin *et al*., 2004). Similarly, it was able to identify that a lower density of homologous genes obtained at the distal end of the 4AL‐TM telosome (Figure 3) is representing the region harbouring the segment introgressed from *Tm*. The *Tm* introgression overlaps with almost the entire chromosome 7BS segment now present on 4AL (Figure 3, Table S2), while the proximal region of the 4AL‐CS and 4AL‐TM telosomes is essentially of bread wheat origin. The number of wheat loci retained in this latter region did, however, differ by 16% in gene content (4AL‐CS—1030 and 4AL‐TM—863 genes). This difference may be result of lower sequencing coverage of the 4AL‐TM (30x compared to 116x of the 4AL‐CS (IWGSC, 2014)) and thus lower representation of the 4AL‐TM sequence assembly. If we assume the similar gene density in homologous chromosomes of relative species, as reported before by Tiwari *et al*. (2015), and if the same rate of missing genes as above due to sequencing and assembly imperfections is assumed, estimated 169 CS nonhomologous genes were carried by the introgression in linkage drag. Knowledgeable selection of parental lines that have relatively high frequency of homologous genes in the region of interest (e.g. QTL for resistance in the *Tm* introgression, Figure 3) may increase chances of unobstructed recombination as was observed in the *QPm‐tut‐4A* locus (Jakobson *et al*., 2012). So, reducing the length of the introgression segment by inducing further rounds of recombination can lessen (or even eliminate) any negative effects of linkage drag. Application of the RICh approach should prove informative regarding the order or frequency of homologous genes of any such selections. Overall, the RICh method offers a robust means of both characterizing chromosome rearrangements and of predicting the gene content of a specific chromosomal region. Recent advances in high‐throughput genotyping permits the elaboration of ever higher density linkage maps (Bellucci *et al*., 2015; Chapman *et al*., 2015; Sorrells *et al*., 2011; Wang *et al*., 2014). The status of chromosome flow sorting is such that almost any wheat chromosome (Tsõmbalova *et al*., 2016) and also chromosomes in many crops (Doležel *et al*., 2014) can now be isolated to a reasonable purity, while the advances in NGS sequencing make RICh widely affordable. These developments should facilitate the preparation of materials needed for applying the RICh approach, thereby offering novel opportunities for a wide range of prebreeding activities, positional cloning, chromatin hybridization and structural studies.

## Experimental procedures

### Plant materials

Grains of the bread wheat ditelosomic CS DT4AL line were provided by Dr. Bikram Gill (KSU, Manhattan, KS), those of the two nullisomic–tetrasomic lines N4AT4B and N4AT4D (Sears and Sears, 1978) by the National BioResource Centre (Kyoto, Japan), those of *Tm* (2*n* = 4*x* = 28, genome formula A^t^A^t^GG) accession K‐46007 by the N.I. Vavilov Institute of Plant Industry (St. Petersburg, Russia). The line denoted DT4AL‐TM was generated from the cross CS DT4AL × 8.1: the line carries 40 bread wheat chromosomes and a pair of 4AL telosomes with the *Tm* introgression (4AL‐TM) and is resistant to powdery mildew (Jakobson *et al*., 2012).

### Flow sorting and amplification of the 4AL telosome carried by 4AL‐TM

Liquid suspensions of mitotic chromosomes were prepared from root tips of 4AL‐TM seedlings as described by (Vrána *et al*., 2000). The telosomes were separated from the rest of the genome by flow sorting, using a FACSAria II SORP flow cytometer and sorter (BD Biosciences, San Jose, CA). The level of contamination within a sorted peak was determined using FISH, based on probes detecting telomeric repeats, the Afa repeat and (GAA)_*n*_, following the methods described by Kubaláková *et al*. (2003). The flow‐sorted 4AL‐TM telosomes were treated with proteinase, after which DNA was extracted using a Millipore Microcon YM‐100 column (www.millipore.com). Chromosomal DNA was MDA amplified using the Illustra GenomiPhi V2 DNA amplification kit (GE Healthcare) as described by Šimková *et al*. (2008).

### Identifying the origin of the introgression segment on the 4AL‐TM telosome

Chromosomes of *T. militinae* were flow sorted as described above. However, prior to flow cytometry, GAA microsatellites on chromosomes were labelled by FITC using FISHIS protocol (Giorgi *et al*., 2013). Bivariate analysis (DNA content/DAPI *vs* GAA/FITC) enabled discrimination of 13 of 14 chromosomes of *T. militinae*. Individual chromosome fractions were sorted into tubes for PCR amplification and onto microscopic slides for identification of sorted chromosomes by FISH. Three markers linked to the *Tm* powdery mildew resistance gene *QPm‐tut‐4A* were used for the selection of the critical cluster: these were the microsatellite *Xgwm160* (Roder *et al*., 1998) and two unpublished, one (*owm72*) amplified by the primer pair 5′‐TGCTTGCTTGTAGATTGTGCA/5′‐CCAGTAAGCTTTGCCGTGTG) and the other (*owm82*) by 5′‐GGGAGAGACGAAAGCAGGTA/5′‐CTTGCATGCACGCCAGAATA. Each 20 μL PCR contained 0.01% (w/v) o‐cresol sulphonephtalein, 1.5% (w/v) sucrose, 0.2 mm dNTP, 0.6 U Taq DNA polymerase and 1 μm of each primer in 10 mm Tris‐HCl/50 mm KCl/1.5 mm MgCl_2_/0.1% (v/v) Triton X‐100. The template comprised about 500 sorted chromosomes. Test reactions were seeded with either 20 ng genomic DNA extracted from CS, *Tm*, N4AT4B or N4AT4D, or with 50 pg of MDA amplified DNA from 4AL‐CS and 4AL‐TM telosomes. The reactions were subjected to an initial denaturation (95 °C/5 min), followed by 40 cycles of 95 °C/30 s, 55 °C/30 s and 72 °C/30 s, and completed with an elongation of 72 °C/10 min. The products were electrophoretically separated through 4% nondenaturing polyacrylamide gels and visualized by EtBr staining. The markers were mapped using a F_2_ population bred from the cross CS × 8.1 (Jakobson *et al*., 2012).

### Sequencing of the 4AL telosome

A CSS assembly of CS chromosome arms 4AS (4AS‐CS) and 4AL (4AL‐CS) were acquired from Internation Wheat Genome Sequencing Consortium (IWGSC, 2014). Two sequencing libraries of DNA amplified from the 4AL‐TM telosome were constructed using a Nextera kit (Illumina, San Diego, CA) with the insert size adjusted to 500 and 1000 bp. The resulting clones were sequenced as paired‐end reads by IGA (Udine, Italy) using a HiSeq 2000 device (Illumina). The 4AL‐TM reads were assembled with SOAPdenovo2 software, applying a range of k‐mers (54–99, with a step size of 3) to select the assembly with the highest coverage and the largest N50. Assembled scaffolds (k‐mer of 69, minimum length 200 bp) were chosen for further analysis (Table 1).

### DArTseq and SNP maps for GenomeZipper construction and validation

A DArTseq consensus map, based on four crosses involving cv. Chinese Spring as a parent has been provided by DArT PL (www.diversityarrays.com). Individual maps were created using DArT PL's OCD MAPPING program (Petroli *et al*., 2012) to order DArTseq and array‐based DArTs. DArT PL's consensus mapping software (Raman *et al*., 2014) was applied to create a consensus map using similar strategy as described in Li *et al*. (2015). Version 3.0 of consensus map with approximately 70 000 markers was used in this study.

A SNP deletion map (Balcárková *et al*., unpublished) was used for validation. Genomic DNAs of a set of 15 chromosome 4A deletion lines (Endo and Gill, 1996) and DNAs amplified from 4AL‐CS and 4AS‐CS chromosome arms as controls were genotyped at USDA‐ARS (Fargo, ND) using a iSelect 90k SNP array (Wang *et al*., 2014) on Infinium platform (Illumina). The raw genotypic data were manually analysed using GenomeStudio V2011.1 software (Illumina).

### Comparative analysis and GenomeZipper analysis

Synteny between related genomic segments was assessed using ChromoWIZ software (Nussbaumer *et al*., 2014). The number of conserved genes present within a series of 0.5‐Mbp genomic windows (window shift 0.1 Mbp) was determined. The consensus chromosome 4A linkage map used as the backbone for the GenomeZipper analysis comprised 1780 DArTseq markers (Table S1). As these sequences are mostly short (69 nt) and few identify coding sequence, they were first aligned to the set of 4A CSS contigs, preserving only those contigs that matched the entire DArTseq marker sequence at a level of at least 98% identity. The retained CSS contigs (‘CSS‐DArTseq markers’) were used for the construction of the zipper, which was subsequently validated against the SNP deletion map (2706 SNPs). Similarly as above, only those 4A CSS contigs that aligned with SNP loci along their entire length (98% identity threshold) were retained. Ordering of the CSS‐DArTseq markers was compared with that ordered by SNPs from the deletion bin map and CSS‐DArTseq markers which do not follow the SNP order were eliminated, and a second version of the zipper was generated using the remaining markers (Table S3). This version was revalidated against the SNP deletion map.

### The RICh approach

To identify introgressed/translocated regions, the final 4A zipper was compared to the complete set of CSS sequences obtained from chromosome arms 4BS, 4BL, 4DS, 4DL, 5BL, 5DL, 7AS and 7DS (IWGSC, 2014). Alignments were performed using the BLAST algorithm (Altschul *et al*., 1990). The BLAST outputs were filtered by applying the following criteria: a minimum identity of either 90% (translocation analysis) or 95% (introgression analysis) and a minimum alignment length of 100 bp. For each comparison, the density of homologous genes was evaluated using a sliding window of eleven genes (five upstream and five downstream), and a segmentation analysis was performed using the R package changepoint v1.1 (Killick and Eckley, 2014), applying the parameter segment neighbourhoods method with a BIC penalty on the mean change. The method allows a statistical detection of gene density changes along the chromosome, corresponding to an increase or decrease in the level of synteny. For translocation events, an increase in synteny level with one group of homoeologs is required, while for an introgression, a loss of orthology is anticipated.

## Conflict of interests

Dr. A Kilian is head of Diversity Arrays Technology Pty Ltd.

## Supporting information


**Figure S1** The flow karyotype of DT4AL‐TM, a bread wheat line ditelosomic for 4AL, the distal portion of which includes a segment translocated from *T. militinae*.

**Figure S2** A comparative analysis of the telosomes 4AL‐CS and 4AL‐TM with the *B. distachyon*, rice and sorghum genomes.

**Figure S3** Refining the robustness of the 4A zipper.

**Table S1** Consensus 4A DArTseq map, based on four independent populations, each involving CS as one parent.

**Table S2** The new 4A zipper, composed of 2132 loci, constructed using the CS‐based 4A specific DArTseq map and validated by reference to SNPs mapped using a panel of deletion lines.

**Table S3** The set of CSS‐DArTseq markers used to construct the new zipper.
