# Supplementary figures and images for: The in silico identification and characterization of a bread wheat/Triticum militinae introgression line
Source: Plant Biotechnol J. 2016 Sep 16;15(2):249–56. doi: 10.1111/pbi.12610 (PMC5259550; doi:10.1111/pbi.12610)

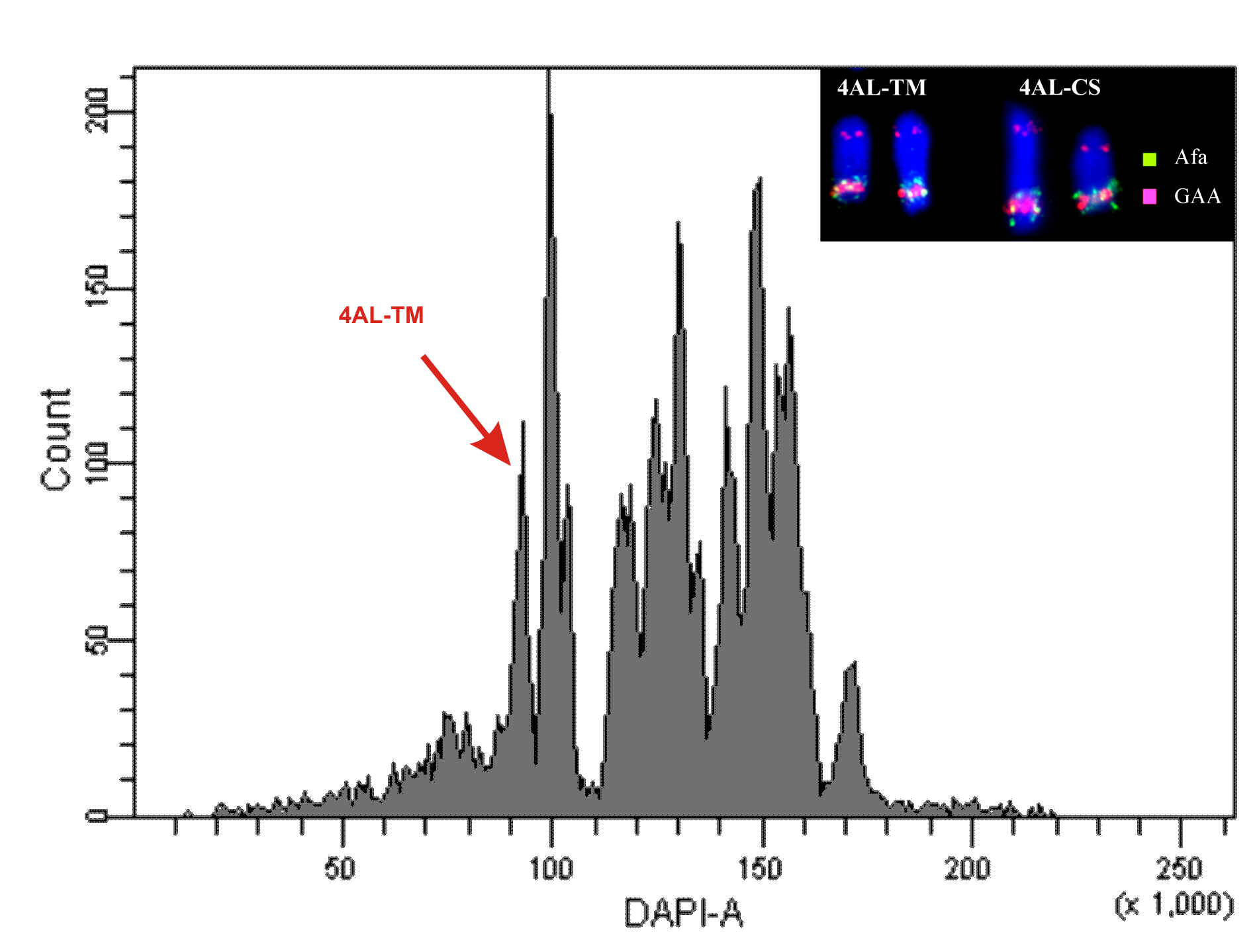

Supplement: Supplementary file 1 — Figure S1 The flow karyotype of DT4AL‐TM, a bread wheat line ditelosomic for 4AL, the distal portion of which includes a segment translocated from T. militinae. [file PBI-15-249-s005.tif]

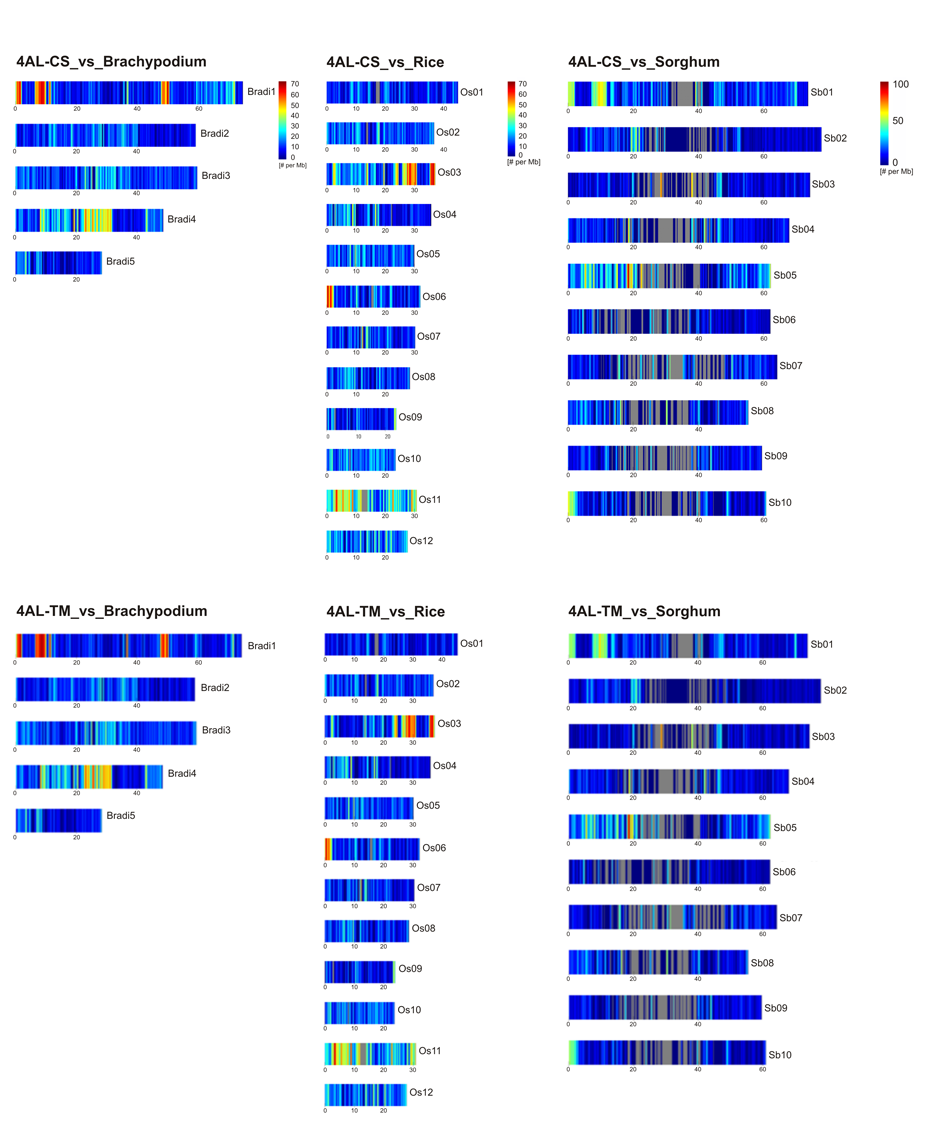

Supplement: Supplementary file 2 — Figure S2 A comparative analysis of the telosomes 4AL‐CS and 4AL‐TM with the B. distachyon, rice and sorghum genomes. [file PBI-15-249-s004.tif]

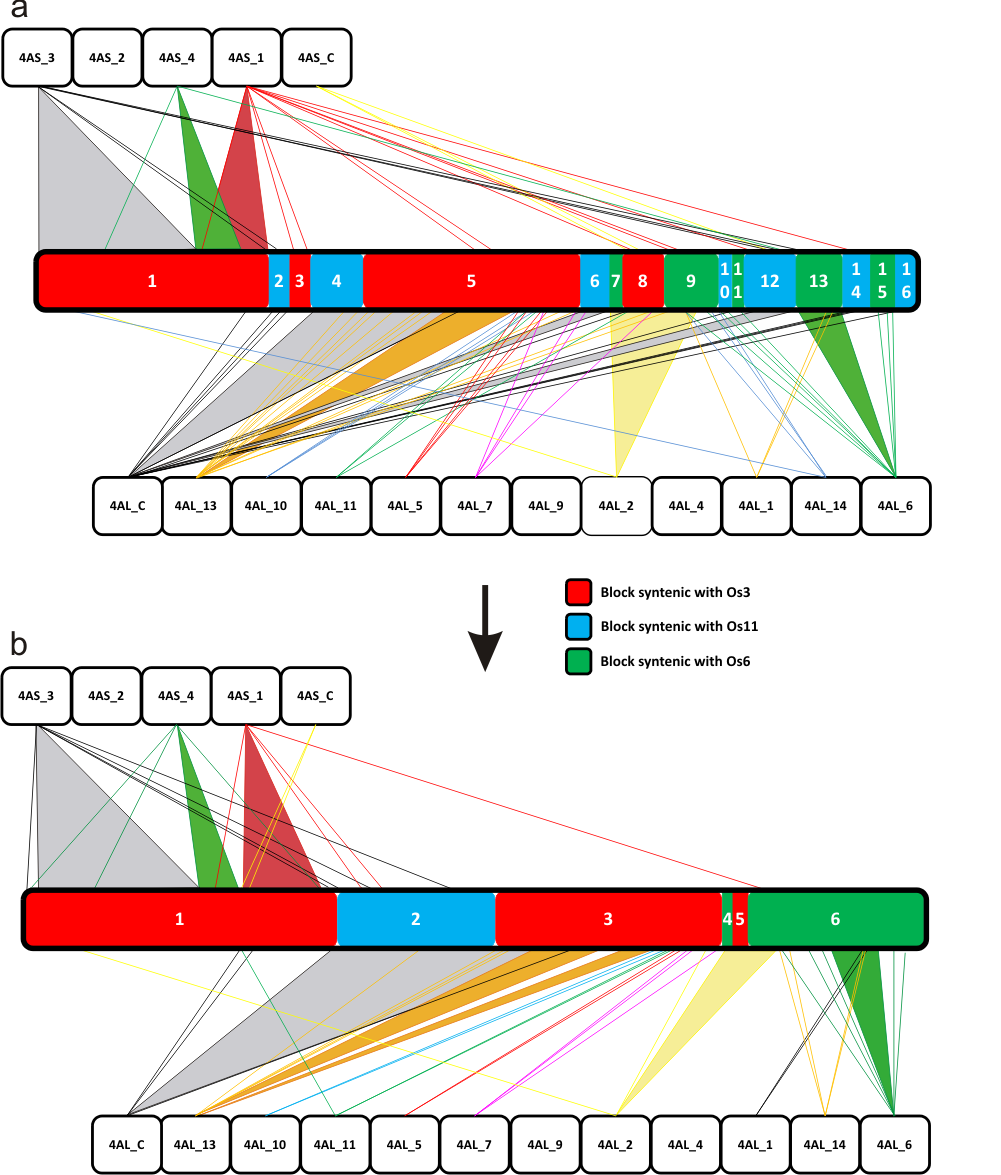

Supplement: Supplementary file 3 — Figure S3 Refining the robustness of the 4A zipper. [file PBI-15-249-s003.tif]
